# Large Cell Calcifying Sertoli Cell Tumor with Macrocalcification in a Partially Resected Testis

**DOI:** 10.1155/2020/5279013

**Published:** 2020-03-18

**Authors:** Mahmoud Bardisi, Mohamad Nidal Khabaz, Jaudah Ahmad Al-Maghrabi

**Affiliations:** ^1^Riyadh Regional Lab and Blood Bank, Ministry of Health, Riyadh, Saudi Arabia; ^2^Department of Pathology, Rabigh Faculty of Medicine, King Abdulaziz University, Jeddah, Saudi Arabia; ^3^Department of Pathology, Faculty of Medicine, King Abdulaziz University, Jeddah, Saudi Arabia

## Abstract

Large cell calcifying Sertoli cell tumors (LCCSCTs) are extremely rare, with less than 100 tumors being described to date. Most of the tumors are benign with a few malignant cases, and aggressive behavior is infrequent. These tumors are a type of Sertoli cell tumor, and these tumors comprise less than 0.3% of all testis tumors in Saudi Arabia. They usually occur in boys and young adults and can affect one or both testicles in multifocal form causing microcalcifications. A 28-year-old male visited our hospital with left testis pain. Physical examination of the scrotum revealed that both testicles were normal sized with no palpable mass. Ultrasonography evaluation revealed grade 3 left varicocele and an incidental 9 mm calcified mass in the right testicle, which was further confirmed by MRI. Partial orchiectomy was performed. Clinical data, radiological studies, and morphological and immunohistochemical characteristics were analyzed.

## 1. Introduction

Sex cord stromal tumors of the testicles (SCSTs) are infrequent and constitute up to 5% of all adult testicle tumors, while the remainder are of germ cell origin [1, 2]. SCSTs are generally benign tumors, but less than 10% of them show malignant features. The main histotypes of these neoplasms are Leydig, Sertoli, and granulosa cell neoplasms. Leydig cell tumors (LCTs) form the bulk of SCSTs and Sertoli cell tumors (SCTs), which are uncommon and comprise less than 1.5% of all testicle neoplasms [1, 2]. Various pathological variants of Sertoli cell tumors were described including LCCSCTs, SCTs not otherwise specified (NOS), sclerosing SCTs, and sex cord with annular tubules. However, the World Health Organization (WHO) recently published an update to the classification of testicular nongerm cell tumors that included some key modifications to the 2004 classification [1–3]. One of the changes related to SCTs was that large cell calcifying Sertoli cell tumor (LCCSCT) has been classified as a separate entity that has different clinical presentation and histopathologic characteristics [1–3].

LCCSCTs are extremely rare tumors. Most of them are benign, but a few malignant cases have been reported [1, 4–6]. They were first identified by Proppe and Scully in 1980, and since then, almost 100 cases have been described in the literature [4, 7–14]. These tumors are usually seen in young adults and can affect one or both testicles in a multifocal form causing microcalcifications [5]. In this study, we describe the clinicopathological data of a 28-year-old male who had a unilateral mass that was incidentally discovered by ultrasonography. Histopathologic examination showed LCCSCT, without alterations in the hormone profile or clinical features of genetic disorders.

## 2. Case Report

**Figure 1:**
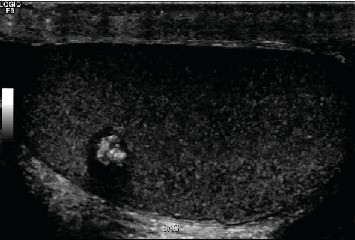
Ultrasound image showing 9 mm right testicular calcified lesion.

**Figure 2:**
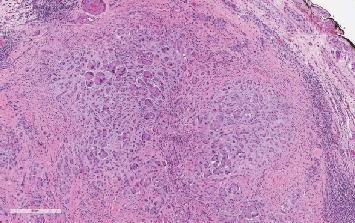
Well-circumscribed mass of LCCSCT (H&E stain; ×10).

**Figure 3:**
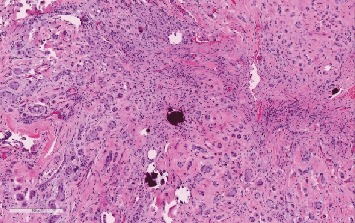
Psammomatous calcification within the neoplastic nests (H&E stain; ×10).

**Figure 4:**
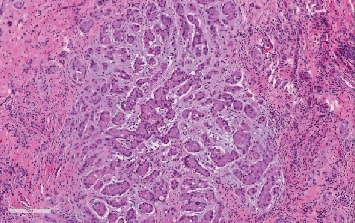
LCCSCT shows a myxoid stromal component (H&E stain; ×20).

**Figure 5:**
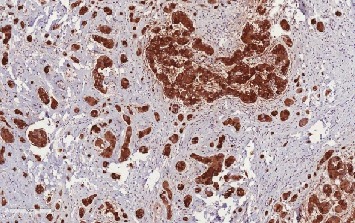
LCCSCT cells show strong inhibin expression (×10).

**Figure 6:**
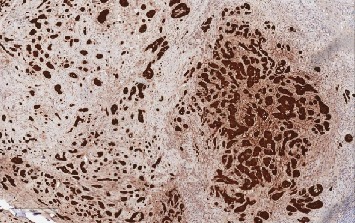
LCCSCT cells show intense calretinin expression (×10).

**Figure 7:**
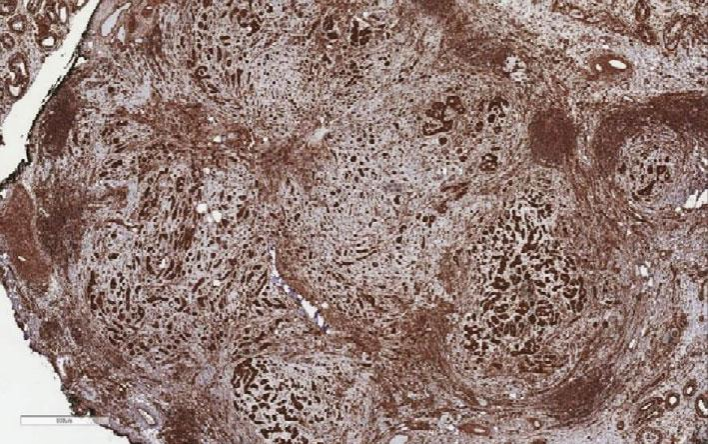
LCCSCT cells show intense vimentin expression (×4).

**Figure 8:**
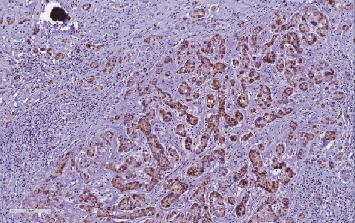
LCCSCT cells show strong Mart-1 expression (×20).

**Figure 9:**
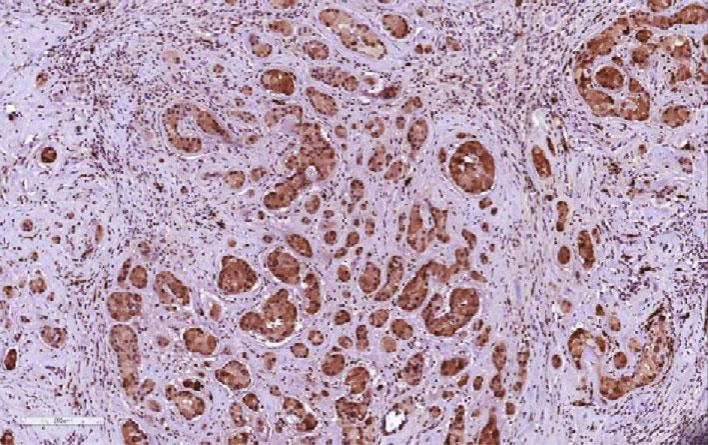
LCCSCT cells show strong S100 expression (×20).

A 28-year-old male attended our hospital complaining of left testicular pain. Physical examination of the scrotum revealed that both testicles were normal in size with no palpable mass. Investigation at the time of admission revealed the following: hemoglobin 157, WBC 7.54, platelet 314, INR 1.1, urea 5.7, creatinine 91, sodium 140, potassium 4.6. AFP 1, HCG less than 2, FSH 3, LH 5, prolactin 7.50, and testosterone 27.0. Semen analysis revealed oligoteratozoospermia. An ultrasonography evaluation revealed grade 3 left varicocele and an incidental 9 mm right testicular calcified lesion (Figure 1). Further clinical examination was unremarkable and revealed neither gynecomastia nor distant metastasis. Human chorionic gonadotropin hormone and *α*-fetoprotein serum levels showed a normal profile. The patient then underwent partial orchiectomy. Gross examination showed fragments of normal-appearing testicular tissue and a well-circumscribed white, firm, round mass measuring 12 mm with a white homogenous cut surface. The specimen was fixed in neutral buffered formalin (10%) embedded in paraffin wax. Tissue blocks were then cut into 4 *μ*m thick sections. Finally, they were stained with H&E. An immunohistochemical study was performed using an automatic immunostainer. The primary antibodies used were inhibin *α*, epithelial membrane antigen (EMA), CD99 (AKA MIC2), calretinin, pan-cytokeratin (AE1/3), S-100, MART-1, synaptophysin, chromogranin, CK8/18, CD10, and vimentin. Histopathological assessment findings showed a well-circumscribed, encapsulated tumor composed of amorphous calcified areas, surrounded by tumor cells (Figure 2). Tumor cells were large and oval to polygonal in shape, with a highly eosinophilic granulated cytoplasm (Figure 3). Tumor cells arranged were in a tubular fashion and surrounded by fibromyxoid stroma that was infiltrated by acute and chronic inflammatory cells. Nuclear pleomorphism was prominent, but mitotic activity was rare and there was no evidence of necrosis (Figure 4). Residual testicular parenchyma was normal, and unremarkable epididymis tissue was observed. Tumor cells were positive for inhibin *α*, epithelial membrane antigen (EMA), calretinin, S-100, MART-1, and vimentin and negative for CD99 (AKA MIC2), pan-cytokeratin (AE1/3), synaptophysin, chromogranin, CK8/18, and CD10 (Figures 5–9). According to the microscopic morphology and immunohistochemical profile, the diagnosis was a “benign large cell calcifying Sertoli cell tumor.” One year later, the patient follow-up has ruled out metastases.

## 3. Discussion

LCCSCT is a rare neoplasm that is a type of Sertoli cell tumor, and it accounts for less than 0.3% of all testis tumors in Saudi Arabia [15]. These tumors usually develop in boys and younger adults, and the majority (80%) are benign. They can occur in one or both testicles in a multifocal pattern, causing microcalcifications [5]. Benign tumors are present mainly in younger patients (early-onset LCCSCTs) and commonly noticed before the age of 17 years, while malignant tumors usually develop in older patients whose average age is 39 years (late-onset LCCSCTs) [4]. According to Kratzer et al. [17], there are some features that may suggest malignant behavior, such as solitary LCCSCT, patient ≥ 25 years of age, size > 4 cm, extratesticular extension, mitotic rates of more than three mitoses per 10 HPFs, coagulative tumor cell necrosis, vascular space invasion, and high-grade cytologic atypia. LCCSCTs are mainly sporadic, which account for 60% of the cases, whereas the remaining 40% may occur as part of autosomal dominant multiple neoplasia syndromes, including the Carney complex (endocrine overactivity, skin pigmentation, and cardiac myxoma), which is marked by a germline PRKAR1A gene mutation, and Peutz–Jeghers syndrome (mucocutaneous pigmentation, gastrointestinal polyposis). They could also be associated with extragonadal endocrine manifestations such as gynecomastia, sexual precocity, and acromegaly [10, 11, 16]. These tumors are usually multifocal and bilateral when they are associated with the above syndromes, while sporadic tumors are most often unilateral and unifocal [2, 5, 7–9, 12].

The patient in the current report had a unilateral and solitary tumor without any evidence of familial syndrome. Among 100 cases, 16 cases of LCCSCT were reported to be malignant [10]. LCCSCT cells are typically organized in the shape of solid tubules, nests, clusters, and cords in a fibromyxoid stroma that often contains neutrophils. The nuclei have a round to oval shape with nucleoli that are small to moderate in size. An intratubular tumor is present in about 40% of the cases. Calcification may vary from a small psammoma body to a prominent plaque-like ossification [1, 9]. Our patient had most of these features; his tumor was a well-circumscribed encapsulated tumor that was composed of amorphous calcified areas, which were surrounded by tumor cells. Neoplastic cells showed similar shape and size as described in the literature. The cells were arranged in a tubular fashion, in a fibromyxoid stroma with acute and chronic inflammatory cell infiltration. Mitotic activity was rare. Nuclear pleomorphism was prominent, and no necrosis was detected.

However, histopathologic differential diagnosis between LCCSCT and large cell hyalinizing Sertoli cell neoplasia is significant and must be considered. The former is most commonly associated with the Carney complex, while the latter is characteristic of Peutz–Jeghers syndrome. However, bilateral, multifocal, intratubular proliferations of large Sertoli cells may develop in both tumor types, but large cell hyalinizing Sertoli cell neoplasia usually shows a greater degree of tubular expansion, deposits in basement membrane are more common, and calcifications are less frequent or less extensive. These patients also less commonly have an invasive tumor at diagnosis [16]. LCCSCT must be distinguished from other more frequently diagnosed tumors like Sertoli cell tumors NOS. These tumors typically consist of clear cells, which often contain lipid-rich vacuoles. Some of these neoplasms contain cells with eosinophilic cytoplasm. Unlike LCCSCT, these tumors lack cells with rich cytoplasm and have no inflammatory cell infiltration and absent laminated calcifications; a multifocal form is infrequent, and there are no accompanying endocrine disorders [18]. LCCSCT should also be differentiated from Leydig cell tumors because both tumors show some similar clinical and histopathologic features such as strong expression of calretinin, inhibin, and MART-1. However, chromogranin is not expressed in either LCCSCT or Leydig cell tumors; synaptophysin is usually negative in LCCSCT, but it may be expressed in LCT (but is uncommon), and S100 is consistent and strong in LCCSCT and weak and variable in LCT (19). The presence of histologic features such as huge calcification or mulberry-like calcification, an intra-tubular development model with thickened basal lamina of these tubules, plentiful myxoid stroma, and inflammatory cell infiltration supports a diagnosis of LCCSCT. In addition, Reinke crystals do not occur in LCCSCT [9, 19, 20], but the presence of calcification in LCCSCT has to be distinguished from those in seminoma, embryonal carcinoma, and teratoma [14].

## 4. Conclusion

The present case is consistent with the previously reported LCCSCT cases. LCCSCTs are very rare tumors. Most of them are benign with a few malignant cases. It is highly recommended that prompt genetic tests and clinical assessments are applied in patients with LCCSCTs that show syndromic features (e.g., typical skin pigmentation, young age, and bilateral/multifocal) to clarify the possible association between these tumors and genetic disorders.
